# Childhood Adversity and Trajectories of Disadvantage Through Adulthood: Findings from the Stockholm Birth Cohort Study

**DOI:** 10.1007/s11205-016-1528-6

**Published:** 2016-12-29

**Authors:** Ylva B. Almquist, Lars Brännström

**Affiliations:** 1 Centre for Health Equity Studies (CHESS), Stockholm University/Karolinska Institutet, 106 91 Stockholm, Sweden; 2 Department of Social Work, Stockholm University, 106 91 Stockholm, Sweden

**Keywords:** Childhood, Disadvantage, Living conditions, Longitudinal, Sequence analysis, Life-course trajectories

## Abstract

Children whose parents experience adverse social, economic, or health-related living conditions are more likely to face similar types of disadvantage in their adult life. However, a limitation of many earlier studies is that they do not account for the multidimensionality of the concept of living conditions, and that the child generation’s life courses are targeted as static and independent from the societal context in which they are imbedded. The current investigation addressed these aspects by focusing on the complexity, duration, and timing of disadvantage with regard to how adverse circumstances in the family of origin are associated with trajectories of social, economic, and health-related living conditions across adulthood. We also examined the role of educational attainment for these associations. Analyses were based a Swedish cohort born in 1953 (n = 14,294). We first conducted sequence analysis, followed by hierarchical cluster analysis, to generate ‘outcome profiles’, i.e. trajectories of adult disadvantage. Second, several indicators of adverse circumstances in childhood were analysed by means of multinominal regression analysis, showing the odds of ending up in the different trajectories. The results indicated that individuals who grew up under adverse conditions were more likely to experience disadvantaged social, economic, and health-related trajectories. This was particularly the case for trajectories characterised by a high degree of complexity, i.e. coexisting disadvantages, and—among men only—by a longer duration of disadvantage. Educational attainment was identified as a powerful mediator, suggesting that efforts to increase equal educational opportunity may be a way of reducing the intergenerational transmission of disadvantage.

## Introduction

While growing up, children incorporate the values and norms of their culture and develop awareness of their own position in society’s overall structure of class and status. This emergent awareness will partly build on the worldview that parents—from their vantage point in the societal hierarchy—convey to the child, thereby also communicating anticipated limits of opportunity. Accordingly, the parents’ living conditions, as reflected in their social, economic, and health-related resources, tend to be transmitted to their children (Grusec and Hastings 2014; Björklund and Jäntti 2009; Coneus and Spiess 2012; Erikson and Goldthorpe 2002; Ermisch et al. 2012). Some children get off to a good start by being born to parents with higher positions in society who may help them to navigate through life. Others are born to less fortunate parents, whose lack of resources may set them on a more disadvantaged journey. At the societal level, these processes cause inequality to be reproduced from one generation to the next. Although the intergenerational reproduction of inequality has been confirmed by much empirical work, a limitation of many studies is that parental living conditions and/or adult disadvantage usually are restricted to a singular characteristic, to multiple indicators of the same dimension, or to indicators across dimensions that are analysed in isolation (Almquist 2015). Moreover, investigations of future living conditions in the child generation typically target outcomes as static, not dynamic, thus failing to account for changes over the life-course (Elder 1994). An additional point concerns the contextualisation of the child generation’s life courses: outcomes are often measured at a single point in time and such analyses are unlikely to capture the link between ‘biography and history’, i.e. the interplay between individual lives, structural contexts, and societal change (Elder et al. 2003).

Using data from a cohort of more than 14,000 individuals who were born in 1953 and resident in the greater metropolitan area of Stockholm, Sweden, the current study intends to fill some of the knowledge gaps identified in previous research. The overall aim is to examine how adverse circumstances in the family of origin relate to the child generation’s own trajectories of social, economic, and health-related disadvantages across adulthood. Instead of targeting only one type of living conditions, we combine indicators across several areas of life. With regard to adverse circumstances in the parental generation, they here encompass alcohol problems, mental health problems, social assistance recipiency, and criminality. Studies have shown that children who experienced these types of problems while growing up, are more likely to suffer from multiple types of disadvantage as adults (Almquist 2015). Concerning disadvantages in the child generation, the current investigation includes three broad indicators of social, economic, and health-related disadvantages: social assistance recipiency, unemployment, and mental health problems. Results from a recent study indicate that the co-occurrence of such problems in midlife is associated with increased risks of premature mortality (Torssander and Almquist 2016). Together with this multidimensional approach, we furthermore incorporate issues of both individual and societal change by focusing on duration (temporary vs. persistent disadvantage) and timing (in relation to macro-level fluctuations) of disadvantage. Apart from analysing these intergenerational associations we will examine the role of a potentially powerful explanatory factor, namely education. In light of the many studies highlighting education as a key element in the intergenerational transmission of resources (d’Addio 2007), it is likely that educational attainment mediates—either fully or in part—the association between adverse living conditions in childhood and adult trajectories of disadvantage. Finally, since men and women have been shown to differ regarding the presence of many types of disadvantage, special attention will be paid to gender.

The remainder of this article is structured as follows: Chap. 2 presents a life-course approach to the intergenerational reproduction of inequality, focusing on the complexity, duration, and timing of disadvantage. In Chap. 3, we describe the data material used in the current study, along with a detailed account of the variables used. Based on the statistical analyses, Chap. 4 reports on the associations between adverse family-related circumstances in childhood and outcome profiles of social, economic, and health-related disadvantages across adulthood. It also entails results from a mediation analysis of educational attainment. Chapter 5, finally, provides a summary of the results along with a discussion of possible implications as well as strengths and limitations of the study.

## A Life-Course Approach to Disadvantage

### Disadvantaged Living Conditions Across the Life Course

While the reproduction of inequality is a macro-level phenomenon, it is driven by patterns of individual development: the conditions under which each adult generation live are at the same time the circumstances that shape the child generation’s life courses. According to the theory of ‘cumulative disadvantage’ (Dannefer 2003; DiPrete and Eirich 2006), adverse circumstances at early stages of life tend to bring about negative trajectories that are not only reflected but perhaps even reinforced across later stages. Empirical evidence for these processes has been found in a vast amount of studies, concluding that children whose parents experience adverse social, economic, or health-related living conditions are more likely to face similar types of disadvantage in their adult life (Björklund and Jäntti 2009; Coneus and Spiess 2012; Erikson and Goldthorpe 2002; Ermisch et al. 2012; Kauppinen et al. 2014).

#### Complexity of Disadvantage

The individual’s overall living conditions reflect her access to resources within multiple areas of society, encompassing e.g. economic capital, labour market attachment, and health (Tåhlin 1990). These resources are interdependent of one another since a disadvantaged situation within one area often occurs simultaneously with the lack of resources in one or more additional areas. For instance, health problems could affect the ability to work whereas unemployment may cause economic hardship. While causal relationships are bound to exist between these different types of disadvantage, the current study makes no a priori assumption about the direction: for example, health problems can both be a cause and a result of economic hardship (Thiede and Traub 1997; Kawachi et al. 2010). Past research has used many different terms to describe the interdependence between areas of living conditions, most of which define it as something that is accumulated and concurrent. In line with some recent studies (Heap et al. 2013; Heap and Fors 2015; Almquist 2015), we use the term ‘coexisting disadvantages’ to highlight these properties. The presence of coexisting disadvantages is not only reflecting a troublesome situation for the individual but could be seen as an indicator of inequality at the societal level since it implies that the distribution of resources is dominated by the same principles across several policy areas (Fritzell and Lundberg 2000). Insofar as the process of cumulative disadvantage is a valid description for how resource deficiencies early in life leave a ‘social imprint’ on a child, thereby making her more vulnerable toward subsequent risk exposures and events (Bäckman and Nilsson 2011; Bäckman and Palme 1998), we assume that adverse family-related circumstances in childhood are related to particularly high odds of experiencing coexisting disadvantages in adult life (see also Almquist 2015).

#### Duration of Disadvantage

Trajectories can be seen as “paths of change in developmental processes” (Van Geert 1994: 31), pointing to the dynamic nature of life courses. Yet, research focusing on intergenerational correlations often measure living conditions at a single point in time. It is reasonable to assume that temporary disadvantage reflects a less severe situation compared to more persistent disadvantage. While this also applies to adverse family-related circumstances in childhood, the current study focuses primarily on the duration of disadvantage in terms of adult outcomes. Previous studies have for example found that parental social assistance receipt is associated not only with the adult children’s social assistance recipiency but also with a longer duration of receipt (Kauppinen et al. 2014). Similar to the reasoning presented regarding complexity of disadvantage, we hypothesise that the general vulnerability of individuals who were exposed to adverse living conditions while growing up increases their likelihood to end up in more persistent (either specific or coexisting) disadvantage rather than shorter periods of single or multiple problems. To the extent that this reflects the failure of institutions to provide the support needed for these individuals to regain good health or to re-enter the labour market, it is poses a problem for society as a whole.

#### Timing of Disadvantage

Human lives are also shaped by questions of “when” and “where”. This is perhaps especially palpable when it comes to societal changes. Our measurement period of disadvantages—the years from 1992 until 2008—is a time period characterized by great macroeconomic dynamics. Before the economic recession hit Sweden in the early 1990s, unemployment rates were exceptionally low—both historically and in comparison with other countries. During the crisis, unemployment levels sky-rocketed from 1.6 to 8.2% where they remained fairly stable until 1997 when they started to fall (Holmlund 2006). In the wake of the recession, the need for cost control led to less generous unemployment benefits. The burst of the dot-com bubble in the early 2000s caused the unemployment levels to rise again. This recession was however limited in scope and the unemployment rate was around 6% when the global financial crises hit Sweden in 2008 (OECD 2016). Unstable conditions at the macro-level are also reflected by downturns in household economies. The 1990s recession caused a rapid increase of social assistance recipiency that has remained at higher levels than before the crises, also after public finances had been restored (Bäckman and Bergmark 2011). Periods of economic recession may, moreover, be particularly difficult for individuals who suffer from mental health problems. This could be due to the increased competitiveness at the labour market where those with poor mental health may not only be at greater risk of losing their job but also be less likely to come back from unemployment spells (Evans-Lacko et al. 2013). The socio-historical context of the 1990s and 2000s thus makes an interesting background for examining whether individuals who come from adverse backgrounds are hit harder by ‘system shocks’, such as deep recessions, compared to others. If so, we expect those who experienced adverse family-related circumstances in childhood to display much higher odds of disadvantage in the early 1990s compared to other specific time periods.

### The Mediating Role of Educational Attainment

Education has been proposed as a key mechanism for the distribution of resources in society. To the extent to which educational opportunity is equal—i.e. that people’s chances of reaching a certain social position in society is a function of their ability and effort, regardless of social background—education would help breaking the intergenerational reproduction of inequality (Rumberger 2010; Breen and Jonsson 2007). Past research has provided mixed evidence for this notion. On the one hand, among individuals who manage to complete a college degree, adverse childhood origins seem to matter less (or not at all) for adult outcomes related to e.g. socioeconomic position (Torche 2011). On the other hand, access to college is highly reliant upon family background in many parts of the world (Haveman and Smeeding 2006). Also in a country such as Sweden, where education is fully subsidized for all citizens, educational skills and ambitions are socially patterned and thereby limit the educational attainment of some groups of individuals. In the current study, we expect that adverse family-related circumstances in childhood are associated with a lower chance of reaching higher levels of education, which in turn would lead to more disadvantaged trajectories of social, economic, and health-related conditions across adulthood. Similar to other studies (Breen and Karlson 2013), we thus treat educational attainment as a possible mediating factor. Although we expect to find strong mediating effects, this will not dismiss education as a (future) promoter of equality: improved educational policy would still have great potential of ameliorating upward mobility and thus change (or break) life-course trajectories of disadvantage.

## Data and Methods

### The Stockholm Birth Cohort Study

The Stockholm Birth Cohort study (SBC) was created in 2004/2005 through a probability matching of two anonymous datasets: the Stockholm Metropolitan Study (SMS) and the Swedish Work and Mortality Database (WMD). The SMS is defined as all children born in 1953 who were living in the greater Stockholm Metropolitan area in 1963 (15,117). The WMD encompasses all individuals in the Swedish population who were alive and resident in Sweden in 1980 and/or 1990. The matching procedure, which was based on an algorithm comparing the SMS and the WMD from a set of variables equal to the two datasets, resulted in 14,294 individuals being included in the SBC (Stenberg and Vågerö 2006; Stenberg et al. 2006).

### Dependent Variables

With regard to social, economic, and health-related disadvantages in adulthood, three indicators were included: social assistance recipiency, unemployment, and mental health problems. All of them covered the period 1992–2008 (ages 39–55).

Information about *social assistance recipiency* was derived from the Longitudinal Integration Database for Health Insurance and Labour Market Studies (LISA), held by Statistics Sweden. In the Swedish case, social assistance recipiency is regulated by national legislation but administered at the municipality level. Since it is a comprehensive means-tested programme all residents who are not able to support themselves financially are eligible for this kind of benefit. For the purpose of the current study, this information was transformed into a dichotomous variable indicating the recipiency of social welfare for each separate year. Also from LISA, information about days in full-time *unemployment* was derived. Similar to social assistance recipiency, a dichotomous variable for each year was created, reflecting any experience of unemployment regardless of duration or frequency. From the Hospital Discharge Register, held by the National Board of Health and Welfare, information about *mental health problems* was gathered. This information was dichotomized to include the occurrence of any hospital discharge due to mental and behavioural disorders. Such disorders include all diagnoses as indicated by codes 290-319 in International Statistical Classification of Diseases and Related Health Problems, Ninth Revision, and codes F00-F99 in International Statistical Classification of Diseases and Related Health Problems, Tenth Revision (however, codes 312-319 and F80-F98 were excluded since they reflect disorders with onset in childhood). Examples of the most common diagnoses are anxiety disorders, major depression, and alcohol and drug dependence.

**Fig. 1:**
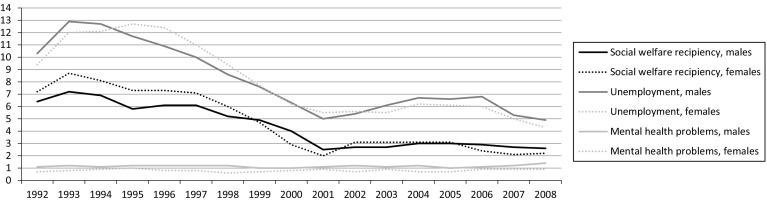
Prevalence (%) of social, economic, and health-related disadvantages in adulthood, 1992–2008 (men, n = 6561; women: n = 6434)

The gender-specific prevalence of the three indicators of disadvantage between 1992 and 2008 can be seen in Fig. 1.

**Fig. 2:**
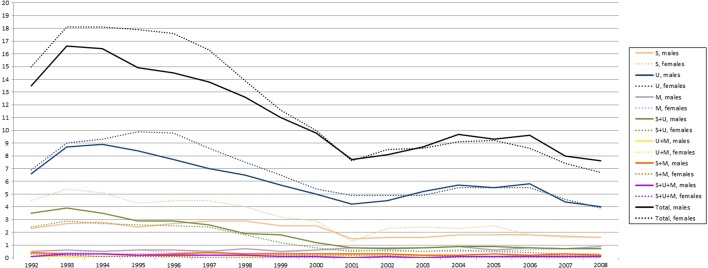
Prevalence (%) of combinations of social, economic, and health-related disadvantages in adulthood, 1992–2008 (men, n = 6561; women: n = 6434). *S* social assistance recipiency, *U* unemployment, *M* mental health problems

A variable with eight categories was created in order to reflect all possible combinations of disadvantage in adulthood. The first category included all individuals who had not experience any of the three types of disadvantage in 1992–2008 (‘No S, U, or M’). The next three reflected social assistance recipiency (‘S’), unemployment (‘U’), and mental health problems (‘M’), respectively. For the subsequent three categories, two-by-two combinations were identified: social assistance recipiency and unemployment (‘S + U’), unemployment and mental health problems (‘U + M’), and social assistance recipiency and mental health problems (‘S + M’). The last category included all three types of disadvantage (‘S + U + M’). Figure 2 shows the gender-specific prevalence of these combinations (except for ‘No S, U or M’) across the period.

### Independent Variables

Four dichotomous indicators of adverse family-related circumstances in childhood were included as independent variables: alcohol problems, mental health problems, social assistance recipiency, and criminality. All of them reflect the period 1953–1972, i.e. when the cohort members were 0–19 years old. The first three types of information were collected from the Social Register and concern both parents, whereas the fourth variable was derived from the National Crime Register and concerns the criminality of the father.

The measure of alcohol problems encompasses cases where either of the parents, or both, was subjected to institutional treatment or action by the temperance committee. Information about mental health problems in the parents reflects any record of psychiatric problems, psychiatric treatment, or suicide. For social assistance recipiency, the information included all income to the family due to such benefits, regardless of duration, amount, or frequency. Concerning the information about criminality of the father, this included all types of sentence, i.e. conditional, unconditional, and exemption from punishment.

Additionally, the analysis included a fifth measure that reflected the number of adverse family-related circumstances in childhood (thus ranging between 0 and 4). It was treated as continuous in the analysis (Table 1).

**Table 1:** Descriptive statistics (%) for the indicators of adverse family-related circumstances in childhood (men, n = 6561; women: n = 6434)

	Men	Women
Adverse family-related circumstances in childhood		
Alcohol problems	4.2	4.1
Mental health problems	6.5	5.9
Social assistance recipiency	21.1	19.4
Criminality	11.7	12.8
Number of adverse circumstances		
0	72.3	73.4
1	16.4	15.4
2	7.8	7.4
3	2.8	3.1
4	0.8	0.7

### Mediating Variables

Information about educational attainment for the year 1990 (age 37) was derived from LISA. The original data, based on the Swedish Educational Terminology (SUN), were collapsed into the following categories: “Compulsory education” (men: 21.8%; women: 15.5%), “Upper secondary education” (men: 42.7%; women: 45.4%), and “University” (men 35.9%; women: 38.8%), Furthermore, an additional category containing those with missing information (men: 0.6%; women: 0.2%) was created.

### Statistical Analysis

Only those who had full information about the study variables were included in the analysis, reducing the original sample from 14,294 to 12,995 individuals (men: n = 6561; women: n = 6434). Missingness was due to lack of information from the LISA registers for one or more of the years 1992–2008, primarily because of death or migration. The analysis was carried out in four subsequent steps, separately for men and women, by means of Stata version 13. The first step was to analyse the trajectories of disadvantage through sequence analysis with the dynamic Hamming algorithm. Next, cluster analysis was applied to identify outcome profiles, i.e. ‘ideal types’ of trajectories. Following this, multinomial regression analysis based on the KHB-method was used to examine how adverse family-related circumstances in childhood related to the risk of ending up in the various outcome profiles, as well as mediation by educational attainment. Each of these steps is described in further detail below.

#### Sequence Analysis

As previously stated, a variable had been constructed reflecting all possible combinations of social assistance recipiency, unemployment, and mental health problems for each year across the period 1992–2008. The categories were ‘No S, U, or M’ (1), ‘S’ (2), ‘U’ (3), ‘M’ (4), ‘S + U’ (5), ‘U + M’ (6), ‘S + M’ (7), and ‘S + U + M’ (8). Based on this information, a 17-state sequence was derived for each individual. This rendered a total of 3064 unique sequences of which the ten most prevalent ones were (in descending order): 11111111111111111, 31111111111111111, 13311111111111111, 13111111111111111, 11111111111111113, 33111111111111111, 21111111111111111, 11131111111111111, 12111111111111111, and 11311111111111111. The first sequence applied to approximately 55.7% of the individuals whereas the remaining covered around 1% each.

The SADI module in Stata was subsequently used to conduct the sequence analysis. Sequence analysis is based on an algorithmic approach that calculates a matrix of dissimilarities (or distances) between pairs of sequences. The most widely used algorithm is the optional matching (OM) algorithm. It operates in three steps—insertion, deletion, and substitution—as a way of expressing distance, i.e. the minimal amount of effort it takes to change two sequences to become identical (Barban and Billari 2012). To each of the steps, a penalty or ‘cost’ is assigned by the researcher. OM has been criticized for difficulties in making theoretically grounded determinations of these costs (Gauthier et al. 2009). The current study therefore used the dynamic Hamming algorithm (Lesnard 2010). This algorithm has been proposed as a potentially better option than OM since it does not require any calculations costs related to insertions or deletions (and, as such, it can only handle sequences of equal length) whereas substation costs are based solely on the data (Aisenbrey and Fasang 2010).

#### Cluster Analysis

Hierarchical cluster analysis was applied on the dissimilarities matrix to group the sequences (the terms cluster and outcome profile will be used interchangeably hereafter). Since conventional test statistics are unavailable for sequence data, the determination of the number of clusters must rely on other arguments. The number of clusters in the present study was determined by the observation of theoretically meaningful clusters, saturation (i.e. when the addition of a new cluster is only a version of an already existing cluster), and a sufficient number of cases in each cluster (Brzinsky-Fay 2007). Based on these principles, we decided to proceed with the eight-cluster solutions for men and for women alike.

#### Multinomial Regression Analysis Based on the KHB-Method

Due to the multi-categorical nature of the outcome, multinomial regression analysis was used to examine the associations between adverse family-related circumstances in childhood and the outcome profiles. The analysis was modelled in two steps: the first model investigated the crude effects between the four separate indicators of family-related circumstances, whereas the second included adjustment for educational attainment. However, logit coefficients from different models are not measured on the same scale and are therefore not directly comparable (Karlson et al. 2012). As a way of dealing with this problem of rescaling, the current study conducted multinomial regression analysis within the framework of KHB (Karlson and Holm 2011). The KHB-method rendered it possible to assess the direct effects of adverse family-related circumstances in childhood on profiles of sequences in adulthood and the indirect effects going through educational attainment (as % reduction).

## Results

### Outcome Profiles of Social, Economic, and Health-Related Disadvantages

**Fig. 3:**
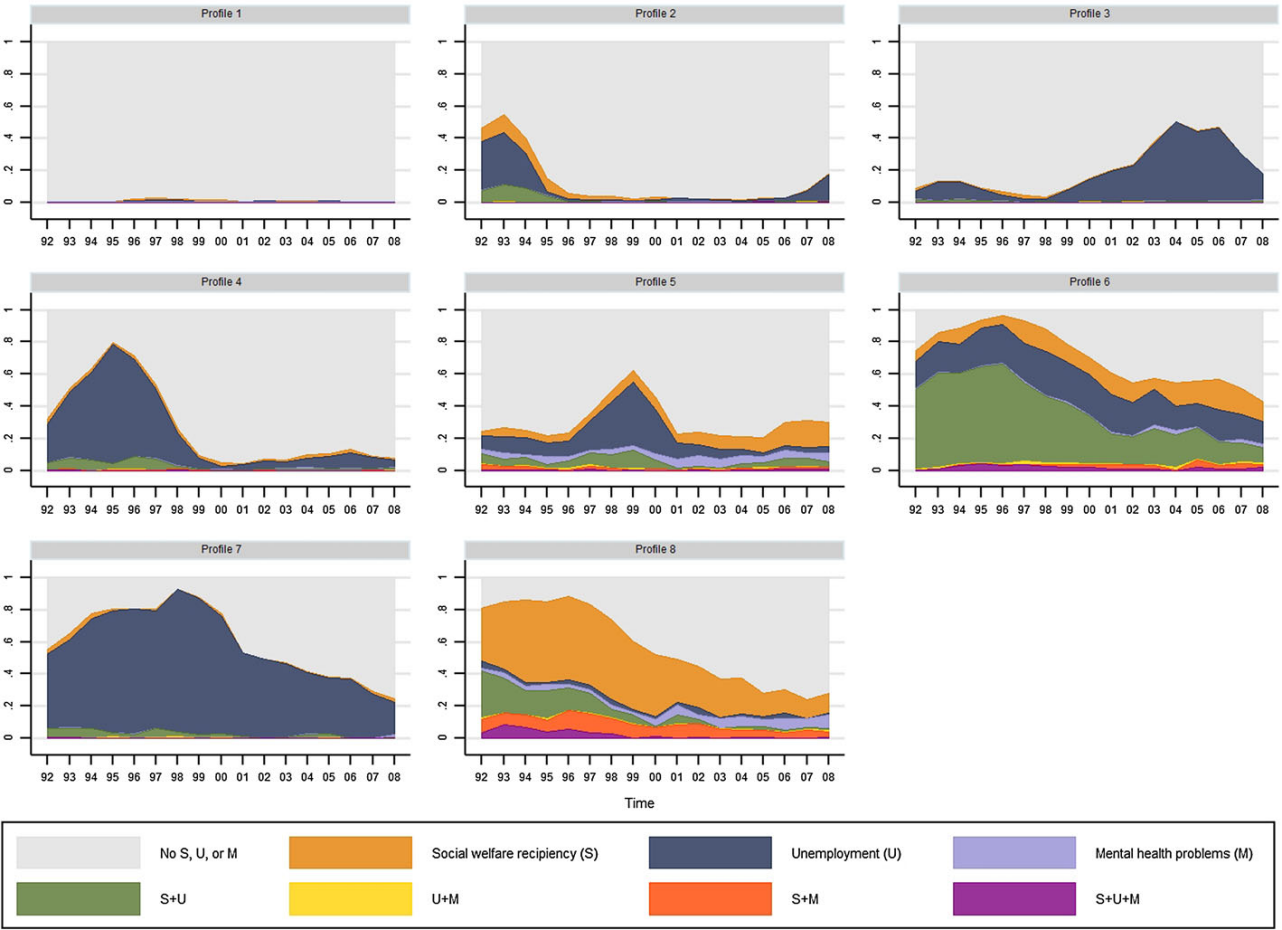
Outcome profiles of sequences reflecting social, economic, and health-related disadvantages across adulthood among men (n = 6561). *S* social assistance recipiency, *U* unemployment, *M* mental health problems. Profile 1: No S, U, or M, 65.4%; Profile 2: S and/or U, early 1990s, 8.5%; Profile 3: U, 2000s, 7.4%; Profile 4: U, 1990s, 6.6%; Profile 5: S, U, and/or M, late 1990s, 5.0%; Profile 6: S and/or U, 2.6%; Profile 7: U, 2.2%; Profile 8: S, U, and/or M, 2.2%

Figure 3 shows the eight outcome profiles of sequences among men. The x-axis reflects the year (1992–2008) whereas the y-axis shows the distribution of the variable indicating combinations of living conditions. Profile 1 shows almost no occurrence of any social assistance recipiency, unemployment, or mental health problems during the period 1992–2008. Hence, it is labelled ‘No S, U, or M’. For Profile 2, there is a peak in the early 1990s with relatively high prevalence of social assistance recipiency and/or unemployment (‘S and/or U, early 1990s’). Profiles 3–4 are characterized by unemployment, where Profile 3 reveals particularly high prevalence across the 2000s (‘U, 2000s’) and Profile 4 has a peak across the 1990s (‘U, 1990s’). Profile 5 has a more complex pattern indicating co-occurrence of social assistance recipiency, unemployment, and mental health problems across the period, with a pronounced peak in the latter half of the 1990s (‘S, U, and/or M, late 1990s’). There is a high prevalence of social assistance recipiency and/or unemployment across the whole period in Profile 6 (‘S and/or U’). The similar pattern is seen in Profile 7, although only for unemployment (‘U’). Finally, Profile 8 demonstrates coexisting prevalence of all three types of ill-fare across the whole period (‘S, U, and/or M’).

**Fig. 4:**
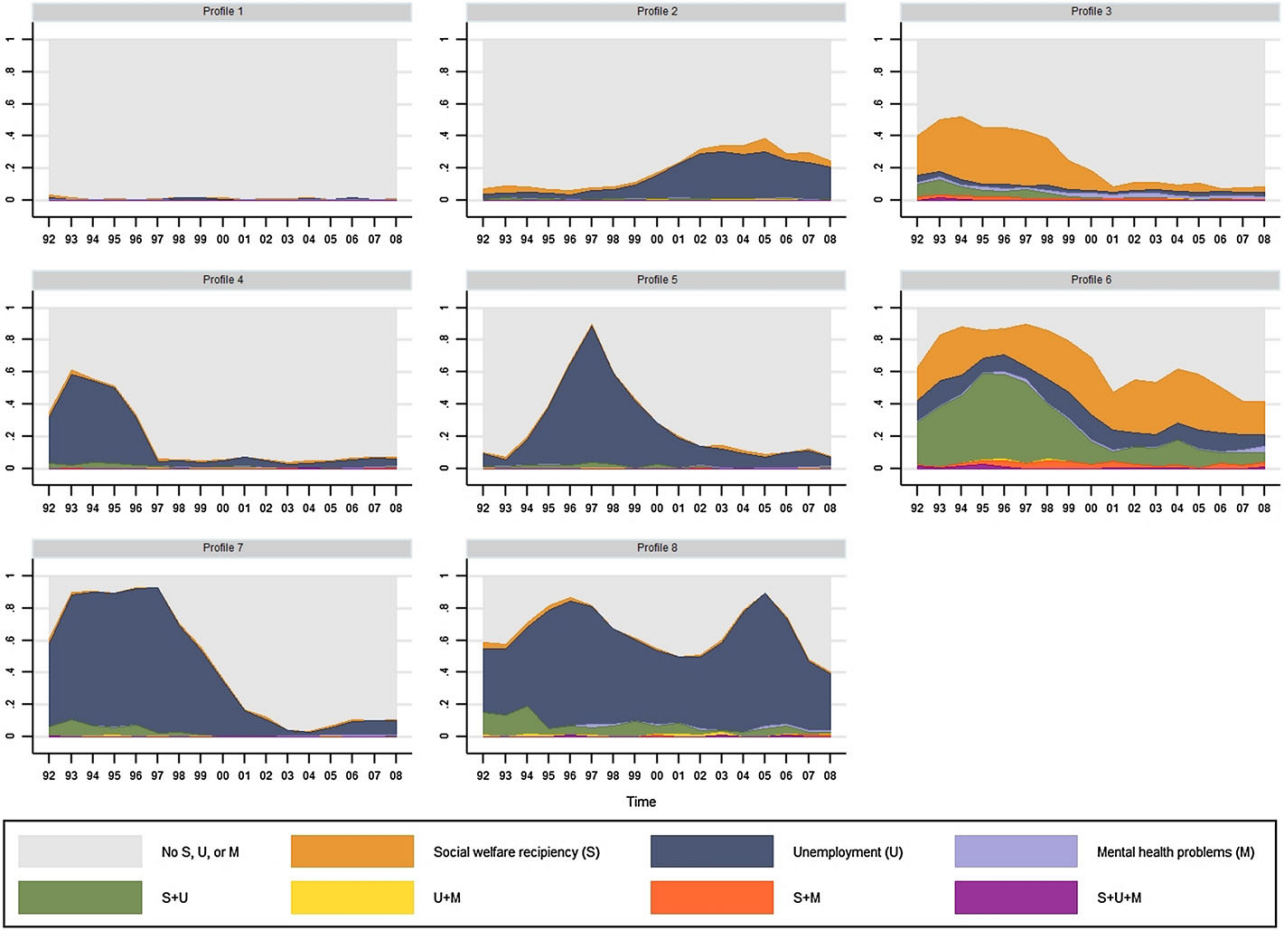
Outcome profiles of sequences reflecting social, economic, and health-related disadvantages across adulthood among women (n = 6434). *S* social assistance recipiency, *U* unemployment, *M* mental health problems. Profile 1: No S, U, or M, 61.1%; Profile 2: U, 2000s, 9.2%; Profile 3: S, U, and/or M, 1990s, 9.1%; Profile 4: U, early 1990s, 8.9%; Profile 5: U, late 1990s, 4.5%; Profile 6: S, U, and/or M, 3.0%; Profile 7: U, 1990s, 2.6%; Profile 8: U, 1.6%

The outcome profiles of sequences reflecting adult living conditions among women can be seen in Fig. 4. Profile 1 shows a cluster where almost no individual had experienced social assistance recipiency, unemployment, or mental health problems in 1992–2008 (‘No S, U, or M’). Profile 2 has high prevalence primarily of unemployment during the 2000s (‘U, 2000s’). In Profile 3, all three indicators of disadvantage are present, particularly across the 1990s (‘S, U, and/or M, 1990s’). The next two profiles—Profiles 4 and 5—are characterized by unemployment. The first of these, Profile 4, has high prevalence of unemployment in the early 1990s (‘U, early 1990s’) whereas the second, Profile 5, shows a peak in the late 1990s (‘U, late 1990s’). All three indicators of disadvantage are present across the entire period in Profile 6 (‘S, U, and/or M’). With regard to the last two profiles, Profile 7 is characterized by unemployment in the 1990s (‘U, 1990s’) whereas Profile 8 has a high level of unemployment throughout the period (‘U’).

### Associations Between Adverse Childhood Conditions and Adult Disadvantage Among Men

Table 2 demonstrate how the four dichotomous indicators of adverse family-related circumstances in childhood are related to the outcome profiles of sequences among men. With regard to parental alcohol problems, the unadjusted results in Model 1 shows that individuals who have experienced such problems in their family of origin, generally have higher odds of ending up in Profiles 2–8, compared to Profile 1. The estimates are however not statistically significant for Profiles 1–2 and 5. Moreover, the odds ratio is particularly pronounced for Profile 6 (OR 5.57), which shows occurrence of social assistance recipiency and/or unemployment across the entire measurement period, and Profile 8 (OR 5.79), which is characterized also by the addition of mental health problems (‘S, U, and/or M’). The inclusion of educational attainment in Model 2 leads to a reduction of 31.2–46.3% of these associations. Despite such large reductions, only the estimate for Profile 4 drops to a statistically non-significant level.

**Table 2:** Adverse family-related circumstances in childhood and outcome profiles of sequences reflecting social, economic, and health-related disadvantages across adulthood, for men (n = 6561)

	Outcome profiles in adulthood (age 39–55)							
	Profile 1	Profile 2	Profile 3	Profile 4	Profile 5	Profile 6	Profile 7	Profile 8
	No S, U or M (ref.), 65.4%	S and/or U, early 1990s, 8.5%	U, 2000s, 7.4%	U, 1990s, 6.6%	S, U, and/or M, late 1990s, 5.0%	S and/or U, 2.6%	U, 2.2%	S, U, and/or M, 2.2%
	OR	OR	OR	OR	OR	OR	OR	OR
Adverse family-related circumstances in childhood (age 0–19)								
Alcohol problems								
Model 1: unadjusted	1.00	1.17	1.51	1.76*	1.69	5.57***	2.06*	5.79***
Model 2: adjusted for educ. attainment	1.00	0.90	1.34	1.36	1.50	2.26***	1.55	3.02***
Mediation effect (% reduction) by educational attainment^a^	–	NA	30.4	46.3	22.9	31.2	39.3	37.1
Mental health problems								
Model 1: unadjusted	1.00	1.66**	0.92	1.01	1.61	3.06***	1.54	3.30***
Model 2: adjusted for educ. attainment	1.00	1.37	0.83	0.83	1.55*	2.04**	1.25	2.02**
Mediation effect (% reduction) by educational attainment^a^	–	37.7	NA	NA	8.3	36.1	49.1	41.1
Social assistance recipiency								
Model 1: unadjusted	1.00	1.63***	1.25	1.50**	1.81	3.40***	1.50*	4.51***
Model 2: adjusted for educ. attainment	1.00	1.29*	1.11	1.17	1.55**	2.07***	1.14	2.49***
Mediation effect (% reduction) by educational attainment^a^	–	48.0	52.7	61.1	25.6	40.5	68.2	39.3
Criminality								
Model 1: unadjusted	1.00	1.50**	1.17	1.53**	1.32	2.24***	1.45	3.87***
Model 2: adjusted for educ. attainment	1.00	1.24	1.06	1.25	1.19	1.46	1.16	2.35***
Mediation effect (% reduction) by educational attainment^a^	–	47.5	64.9	48.0	37.3	52.9	60.3	37.0
Number of adverse circumstances								
Model 1: unadjusted	1.00	1.28***	1.11	1.23**	1.32	1.90***	1.28*	2.19***
Model 2: adjusted for educ. attainment	1.00	1.14*	1.04	1.09	1.23**	1.48***	1.11	1.62***
Mediation effect (% reduction) by educational attainment^a^	–	48.7	60.6	60.6	24.3	39.3	56.0	38.4

Results from multinomial regression analysis based on the KHB-method, presented as odds ratios (ORs)

*S* social assistance recipiency, *U* unemployment, *M* mental health problems, *NA* not applicable, due to reverse change

* p < 0.05; ** *p* < 0.01; *** *p* < 0.001

^a^Decomposition of direct and indirect effects using the KHB-method

Concerning parental mental health problems, Model 1 demonstrates statistically significantly higher odds of ending up in Profiles 2, 6, and 8, with estimates being particularly high for the latter two (ORs 1.66, 3.06, and 3.30, respectively). Thus, while the overall picture is similar to the one present for alcohol problems, the differences are considerably smaller. Educational attainment, which is adjusted for in Model 2, leads to 36.1–41.1% reduction of the strength of these associations. Same as before, however, the increased odds of ending up in Profiles 6 and 8 among those whose parents had mental health problems are not fully accounted for by educational attainment (while the odds for Profile 2 is).

The odds of ending up in Profiles 2–8 compared to Profile 1 are higher among those who grew up in a family that received social assistance recipiency. The estimates for Profiles 3 and 5 fail, however, to reach a statistically significant level. With regard to Profiles 6 and 8, the estimates (ORs 3.40 and 4.51, respectively) are much stronger as compared to most other profiles. Model 2 shows a 39.3–61.1% reduction of these associations by educational attainment. Despite heavy reductions, the estimates for Profiles 2, 6, and 8, remain statistically significant.

The same pattern as for social assistance recipiency is seen for parental (father’s) criminality: the estimates for Profiles 3 and 7 are statistically non-significant while the highest odds ratios are found for Profiles 6 (OR 2.24) and 8 (OR 3.87). According to Model 2, educational attainment accounts for a 37.0–52.9% decrease of these estimates and reduced all estimates expect the one for Profile 8 to non-significant levels.

Overall, examining how the number of adverse circumstances is related to the outcome profiles reveals the similar results compared to the ones for the dichotomous indicators: individuals who had a more adverse upbringing are generally more likely to end up in Profiles 2–8. These associations are particularly evident for the profiles characterized by social assistance recipiency and unemployment in the early 1990s (Profile 2), as well as social assistance recipiency, unemployment, and/or mental health problems throughout the period (Profile 8), or restricted to the late 1990s (Profile 5). Due to the non-linearity of the effects (data not presented), the odds ratios are lower compared to those for the dichotomous indicators. Adjusting for educational attainment lowers the odds ratios with 38.4–48.7% but does not fully explain the associations.

### Associations Between Adverse Childhood Conditions and Adult Disadvantage Among Women

The corresponding results for women are shown in Table 3. With regard to alcohol problems in the family of origin, there are increased risks of ending up in Profiles 2–8 as compared to Profile 1, but only the estimates for Profile 3 (OR 3.04), which is characterized by social assistance recipiency, unemployment, and/or mental health problems in the 1990s, and Profile 6 (OR 6.28), characterized by all three indicators of disadvantage throughout the period, show statistically significant differences. Educational attainment, which is adjusted for in Model 2, leads to 29.2–30.8% reduction of these estimates. However, educational attainment does not fully explain the elevated odds ratios for Profiles 3 and 6.

**Table 3:** Adverse family-related circumstances in childhood and outcome profiles of sequences reflecting social, economic, and health-related disadvantages across adulthood, for women (n = 6434)

	Outcome profiles in adulthood (ages 39–55)							
	Profile 1	Profile 2	Profile 3	Profile 4	Profile 5	Profile 6	Profile 7	Profile 8
	No S, U or M (ref.), 61.1%	U, 2000s, 9.2%	S, U, and/or M, 1990s, 9.1%	U, early 1990s, 8.9%	U, late 1990s, 4.5%	S, U, and/or M, 3.0%	U, 1990s, 2.6%	U, 1.6%
	OR (95% CI)	OR (95% CI)	OR (95% CI)	OR (95% CI)	OR (95% CI)	OR (95% CI)	OR (95% CI)	OR (95% CI)
Adverse family-related circumstances in childhood (ages 0–19)								
Alcohol problems								
Model 1: unadjusted	1.00	1.53	3.04***	1.41	1.50	6.28***	1.29	1.76
Model 2: adjusted for educ. attainment	1.00	1.20	2.20***	1.19	1.21	3.56***	1.00	1.38
Mediation effect (% reduction) by educational attainment^a^	–	56.5	29.2	49.1	53.3	30.8	100.0	43.2
Mental health problems								
Model 1: unadjusted	1.00	1.57	2.34***	0.86	1.69	4.59***	1.30	1.95
Model 2: adjusted for educ. attainment	1.00	1.34	1.82***	0.76	1.48	2.97***	1.11	1.66
Mediation effect (% reduction) by educational attainment^a^	–	35.2	29.4	NA	24.8	28.6	60.8	23.9
Social assistance recipiency								
Model 1: unadjusted	1.00	1.31	3.02***	1.32*	1.41	4.78***	1.68	1.71
Model 2: adjusted for educ. attainment	1.00	1.09	2.22***	1.11	1.21	2.75***	1.40	1.42
Mediation effect (% reduction) by educational attainment^a^	–	69.9	27.7	62.9	44.0	35.4	34.6	34.1
Criminality								
Model 1: unadjusted	1.00	1.50**	1.17	1.53**	1.32	2.24***	1.45	3.87***
Model 2: adjusted for educ. attainment	1.00	1.24	1.06	1.25	1.19	1.46	1.16	2.35***
Mediation effect (% reduction) by educational attainment^a^	–	47.5	64.9	48.0	37.3	52.9	60.3	37.0
Number of adverse circumstances								
Model 1: unadjusted	1.00	1.19	1.70***	1.11	1.25	2.15***	1.22	1.30
Model 2: adjusted for educ. attainment	1.00	1.08	1.46***	1.02	1.15	1.64***	1.11	1.19
Mediation effect (% reduction) by educational attainment^a^	–	55.5	28.3	82.7	35.5	35.5	48.3	36.0

Results from multinomial regression analysis based on the KHB-method, presented as odds ratios (ORs)

*S* social assistance recipiency, *U* unemployment, *M* mental health problems, *NA* not applicable, due to reverse change

* p < 0.05; ** *p* < 0.01; *** *p* < 0.001

^a^Decomposition of direct and indirect effects using the KHB-method

For parental mental health problems, the estimates are much in line with those for alcohol parental problems: the odds ratios are higher for all profiles (expect Profile 4) compared to Profile 1, but only those for Profiles 3 and 5 reach statistically significant levels. While the inclusion of educational attainment in Model 2 causes 28.6–29.4% reduction of these associations, they remain statistically significant.

The similar pattern is shown for those whose family received social assistance recipiency during upbringing, where only the odds of for Profile 3 (OR 2.31), Profile 4 (OR 1.32), and Profile 6 (OR 2.57) are significantly higher compared to the remaining profiles. When educational attainment is taken into consideration, 27.7–62.9% of these associations are accounted for. While the estimates for Profiles 3 and 6 remain at statistically significant levels, the increased odds reported for Profile 4 is fully explained.

Women whose father had any criminal convictions have higher risks of ending up in Profiles 2–8 but, again, only the estimates for Profiles 3 and 6 reach statistically significant levels. Educational attainment accounts for 32.1–51.2% reduction but the associations remain statistically significant.

The fifth indicator—number of adverse circumstances—shows that individuals, who had more adverse circumstances while growing up, have increased odds of ending up in Profiles 2–8 as compared to Profile 1. This is particularly the case for the profiles characterized by social assistance recipiency, unemployment, and/or mental health problems throughout the period (Profile 6), or restricted to the 1990s (Profile 3). Educational attainment seems to be an important mediator, leading to 28.3–35.5% reduction, but the associations remain statistically significant.

## Discussion

The aim of this study was to examine how adverse circumstances in the family of origin relate to the child generation’s trajectories of social, economic, and health-related disadvantages across adulthood. In contrast to many other studies, that have analysed living conditions in isolation from one another, the present study focused on the concept of coexisting disadvantages to capture a more comprehensive picture of adult living conditions. Apart from the issue of complexity, the use of sequence analysis rendered it possible to investigate aspects that concerned both duration and timing of disadvantage across adulthood.

In line with the results from a recently published study that examined the same type of disadvantages between 1992 and 1999 in relation to mortality (Torssander and Almquist 2016), the vast majority of the individuals examined here had no or limited experiences of social assistance, unemployment, and mental health problems. Regarding the remaining outcome profiles (i.e. ‘ideal types’ of sequence clusters), most were characterised by temporary or persistent unemployment. In the current study, about 18% of the men and roughly 12% of the women were found in profiles with coexisting disadvantages, to be compared with 15% of men and 21% of women in the study by Torssander and Almquist (2016). The differences in these rates can most likely be attributed to the differential measurement periods.

The pattern of associations between childhood conditions and the outcome profiles was found to differ between men and women. For men, the highest relative differences were found for the outcome profiles characterised by the coexistence of social assistance recipiency and unemployment—with or without mental health problems. Thus, adverse family-related circumstances seem to lead to a situation characterised by complex and enduring disadvantage in adulthood, not peaking during any specific time period. Among women, parental disadvantages showed the strongest associations with the outcome profiles that reflected the simultaneous occurrence of social assistance recipiency, unemployment, and mental health problems, either across the entire measurement period or limited to the 1990s. Thus, in correspondence with earlier studies showing that adverse family-related circumstances are positively associated with coexisting disadvantage (Almquist 2015), complexity seems to be a highly relevant issue. Duration and timing, however, appear to play less significant roles.

Although implicitly stated above, it is worth highlighting that few (statistically significant) elevated odds ratios were found for outcome profiles indicating single disadvantages. This can be seen as confirming the importance of considering the multidimensionality of living conditions; analysing outcomes in isolation from one another might disguise the presence of heavily problem-burdened groups in society.

It should also be mentioned that the overall patterns of associations were similar across the four indicators of adverse family-related circumstances in childhood as well as the summary measure indicating the number of adverse circumstances. Looking at the size of the odds ratios, however, the relative differences were slightly larger among individuals whose parents had alcohol problems, followed by those whose father had been convicted for a crime. For parental alcohol problems, the connection to outcome profiles involving hospitalisations due to mental health problems is not surprising since these involve substance abuse, thus reflecting the robust intergenerational correlations found in past research (Campbell and Oei 2013). Also with regard to criminality, previous studies have confirmed the strong ‘inheritability’ across generations (Besemer and Farrington 2012). Considering that adult criminality, in turn, is closely correlated with many types of disadvantage such as unstable labour market attachment and disability pension (Nilsson and Estrada 2011), this may explain the strong associations between father’s criminality and the child’s increased odds of ending up in highly problem-burdened outcome profiles.

Moreover, education was analysed as a potentially important factor in terms of linking childhood conditions with later-life trajectories. In line with our initial hypothesis, educational attainment mediated a substantial part of the associations, with some variations across the indicators of diverse family-related circumstances in childhood as well as the outcome profiles in adulthood.

Although educational attainment did not fully account for the associations, it is difficult to imagine another single factor with this much explanatory power. It is possible that a mix of e.g. personality (Groves 2005), behaviours (Duncan et al. 2012), and social networks (Li et al. 2008) could contribute to parts of the explanation.

A major strength of the current study was the use of the Stockholm Birth Cohort study with its detailed and high-quality register-based information about the cohort members’ living conditions from birth up until their mid-50s. Some limitations should however be addressed. To begin with, the measurement period for adverse family-related circumstances in childhood started at birth and ended at age 19. It is possible that adverse living conditions at certain stages of life may affect the child more profoundly—or differently—than other stages. For example, parental alcohol or mental problems very early in a child’s life may cause bigger disruptions in parent–child relationship compared to parental problems first occurring in adolescence. On the other hand, economic hardship or criminality in the family may have a larger impact once the child starts school and acceptance by peers becomes increasingly important. The measurement period of adult disadvantage also encompassed nearly two decades of the cohort members’ lives. Although the years 1992–2008 covered great macro-level changes (e.g. the economic recessions), they represent a relatively stable part of the cohort’s adulthood (ages 39–55) that is largely characterised by stable family and work conditions. However, had it not been for the current data restrictions, it would have been interesting to expand this measurement period to also encompass young adulthood as well as the years up until retirement age.

Register-based information has some inherent weaknesses. For example, it tends to give an ‘aerial view’ of life-course processes since it seldom covers living conditions of psychosocial character, such as social support, working conditions, or quality of life. Moreover, this study was not able to capitalise on register data from other potentially important life domains, reflecting e.g. housing conditions or exposure to crime. The four indicators of adverse family-related circumstances in childhood as well as the three types of disadvantage in adulthood were all dichotomised, thus indicating only their absence or presence. It is possible that more detailed measures had been better at capturing the ‘severity’ of disadvantage. For example, growing up in a family who temporarily received social assistance may not be as influential for subsequent disadvantage in comparison to long-term social assistance recipiency (Wagmiller et al. 2006). Likewise, having had repeated hospitalisations due to mental health problems during a year is likely to reflect a more adverse situation compared to a single hospital stay. Using such detailed -information would nonetheless have increased the number of possible combinations of disadvantage, hampering the interpretation of associations across the outcome profiles. Furthermore, as indicated by the prevalence of the different family-related circumstances, some of the exposures were less common and potentially reflecting a higher degree of severity: e.g. the prevalence of parental alcohol problems was about 4% while approximately 20% grew in families that received social assistance. The same applies to disadvantages in adulthood: whereas approximately 1% of the individuals were hospitalised due to mental health problems between 1992 and 2009, the unemployment rate was comparably higher (about 4–13% across the measurement period). Additional shortcomings are related to the data registers from which the measures of adverse family-related circumstances in childhood were derived. For instance, information about criminality was available for the father only. Moreover, information about alcohol problems, mental health problems, and social assistance recipiency was derived from the Social Register. One potential problem here is, on the one hand, that individuals who came to the attention of the social services for any reason were more likely to be registered also for other reasons. On the other hand, individuals whose problems were not severe or visible enough to be noted by the social services would not show up in the registers at all.

## Conclusions

In sum, the current study found adverse family-related circumstances to be associated with increased odds of experiencing disadvantaged social, economic, and health-related trajectories across adulthood. This was particularly the case for trajectories characterised by a high degree of complexity, i.e. coexisting disadvantages, and—among men only—by a longer duration of disadvantage. These results are thus in line with theories of cumulative disadvantage, reflecting that an adverse situation in childhood may be a marker for accumulated and, to some extent, persistent problems later in life. The fact that educational attainment was shown to be such a powerful mediator suggests that efforts to increase the equality of educational opportunity may have great potential when it comes to reducing the intergenerational transmission of disadvantage.
